# Magnetic resonance imaging improves the prediction of tumor staging in localized prostate cancer

**DOI:** 10.1007/s00261-020-02913-9

**Published:** 2021-01-16

**Authors:** B. Valentin, L. Schimmöller, T. Ullrich, M. Klingebiel, D. Demetrescu, L. M. Sawicki, J. Lakes, D. Mally, M. Quentin, I. Esposito, P. Albers, G. Antoch, C. Arsov

**Affiliations:** 1 Department of Diagnostic and Interventional Radiology, Medical Faculty, University Dusseldorf, Moorenstr. 5, 40225 Dusseldorf, Germany; 2 Department of Urology, Medical Faculty, University Dusseldorf, Moorenstr. 5, 40225 Dusseldorf, Germany; 3 Department of Pathology, Medical Faculty, University Dusseldorf, 40225 Dusseldorf, Germany

**Keywords:** Prostate MRI, Staging, Prostate cancer, PI-RADS, Radical prostatectomy

## Abstract

**Objectives:**

The aim of this study was to investigate 3 Tesla multiparametric magnetic resonance imaging (mpMRI)-based predictors for the pretherapeutic T staging of prostate cancer and their accuracy.

**Methods:**

Consecutive patients with 3 Tesla mpMRI, positive systematic and MR-targeted biopsy, and subsequent radical prostatectomy (RPE) between 01/2016 and 12/2017 were included. MRI parameters such as measurable extraprostatic extension (EPE) (≥ 3 mm), length of (pseudo)capsular contact (LCC), invasion of neurovascular bundle (NVBI), and/or seminal vesicles lesion contact (SVC) or infiltration (SVI) were assessed and correlated to clinical and histopathological results.

**Results:**

136 men were included. In 76 cases, a pT2 stage was determined, in 29 cases a pT3a, and in 31 a pT3b stage. The positive and negative predictive values (PPV, NPV) for the detection of T3 by measurable EPE on MRI was 98% (CI 0.88–1) and 81% (CI 0.72–0.87). No visible NVBI was found in pT2 patients (NPV 100%; CI 0.95–1). ROC analysis for T3a prediction with LCC (AUC 0.81) showed a sensitivity of 87% and a specificity of 62% at a threshold of 12.5 mm (*J* = 0.485) and 93% and 58% at 11 mm (*J*_max_ = 0.512). All patients with pT3a had a LCC > 5 mm. In case of pT3b, 29/31 patients showed a SVC (PPV 76%, CI 0.61–0.87; NPV 98%, CI 0.93–0.99), and 23/31 patients showed a SVI (PPV 100%, CI 0.86–1; NPV 93%, CI 0.87–0.96). EPE (*p* < 0.01), LCC (*p* = 0.05), and SVC (*p* = 0.01) were independent predictors of pT3.

**Conclusions:**

MRI-measurable EPE, LCC, and SVC were reliable, independent, preoperative predictors for a histopathological T3 stage. A LCC ≥ 11 mm indicated a pT3a stage, whereas a LCC < 5 mm excluded it. On MRI, visible SVI or even SVC of the PCa lesion was reliable preoperative predictors for a pT3b stage.

**Supplementary information:**

The online version of this article (10.1007/s00261-020-02913-9) contains supplementary material, which is available to authorized users.

## Introduction

In recent years, the use of multiparametric magnetic resonance imaging (mpMRI) of the prostate has been expanded significantly from not only cancer detection and MRI-based targeted biopsy strategies but also to MRI-based monitoring of low-risk cancers on active surveillance and local staging [1–5]. For appropriate therapy planning, it is important to determine the exact tumor stage as several options including surgery, hormonal therapy, and radiotherapy exist depending on the local cancer growth and spread. Therefore, clinical parameters such as PSA or PSAD are taken into account and predictive models as, for example, the Cancer of the Prostate Risk Assessment (CAPRA) score is used to evaluate the individual risk for PCA patients [6–8]. Ideally, the imaging and preoperative biopsy results should be able to predict the final histopathological tumor stage. Therefore, parameters such as extraprostatic extensions (EPE), focal/microscopic extraprostatic extensions (FEPE, measurable exceedance < 0.1 mm), and seminal vesicle infiltration (SVI) are of particular interest [7]. FEPE can be determined histopathologically and can be defined as single tumor involving glands outside the prostate pseudocapsule in one or two slides with a measurable lengths of < 0.1 mm [9, 10]. Indirect signs, such as extended length of pseudocapsular contact or a protrusion of the pseudocapsule, may indicate microscopic EPE [11]. This means that a pT3a diagnosis is already present if the pseudocapsule is exceeded with single detectable tumor glands in the fatty tissue including FEPE. Thus, exact description and localization of potentially existing EPE and SVI are putatively helpful for the surgeon to prevent remaining tumor residuals (R1 resection). A pathological T3a as well as a T3b stage usually requires adjuvant radiotherapy after surgery. On the other hand, patients without high risk of EPE can potentially be offered a nerve-sparing surgery [12]. In its current version of the Prostate Cancer Guideline, the European Society of Uroradiology (ESUR) describes mpMRI as the currently best available imaging tool for assessing EPE [13, 14]. The use of 1.5 Tesla mpMRI showed a high specificity, but a low sensitivity for the detection of a T3 stage, which may be improved by using an endorectal coil (ERC) [15]. Using 3 Tesla mpMRI, an increase in sensitivity for the detection of a T3 stage has been reported mostly using a surface coil [14]. Currently, measurable or visible EPE and/or SVI are used as predictors for T stage determination on MRI [16]. In addition, the probability of EPE increases with larger capsule contact length (LCC) [17]. Grivas et al. as well as Roethke et al. were able to show that 3 Tesla mpMRI can achieve satisfactory accuracy in the detection of SVI [18, 19]. Nevertheless, the detection of EPE is still challenging.

The aim of this study was to determine the T stage accuracy of high-quality 3 Tesla mpMRI and to find reliable MRI-based predictors for pretherapeutic T staging.

## Methods

### Study design

Consecutive patients with 3 Tesla mpMRI of the prostate, positive systematic plus targeted MR/US-guided fusion biopsy, and subsequent radical prostatectomy (RPE) between 01/2016 and 12/2017 were included in this study. Study approval of the local ethic committee (Medical Faculty of the Heinrich-Heine-University Düsseldorf; Study-ID: 2018084786) exists, and written informed consent was obtained from all patients. Clinical parameters such as age, prostatic-specific antigen (PSA) value, PSA density (PSAD), and ISUP Grade Group as well as MRI-based parameters like PI-RADS classification, EPE, LCC, SVI, or tumor contact with SV (SVC) were assessed and correlated with the histopathologic results after RPE to quantify the impact on T stage prediction of each descriptor.

### MR imaging

MpMRI was performed at a 3 Tesla MRI scanner (Magnetom Trio TIM or Skyra System, Siemens Healthcare GmbH) using an 18-channel phased-array surface coil combined with a 32-channel spine coil. According to the PI-RADS, all protocols included T1WI, T2WI, DWI sequences, as well as dynamic contrast sequences [20]. Sequence parameters are shown in Supplementary Table 1. All patients received butylscopolamine (20 mg Buscopan^®^, Boehringer Ingelheim Pharma) to suppress bowel peristalsis. The classification according to PI-RADS version 2.1 was applied, retrospectively; the length of contact of PCA lesions to the pseudocapsular by LCC (in mm), the invasion of the neurovascular bundle (NVBI), infiltration of the extra periprostatic tissue (EPE), SVI, and SVC of the PCA lesion were determined on MRI by experienced radiologists in consensus (L.S., T.U., and B.V. with 10, 5, and 2 years’ experience in reading prostate MRI). LCC was determined by the greatest extension in coronary, sagittal, or axial T2W sequences. EPE was defined as measurable extension of the tumor over the prostate pseudocapsule of ≥ 3 mm. A new or existing asymmetry of the NVB, an accompanying protrusion of the pseudocapsule, and/or a visible pseudocapsule protrusion with detectable tumor growing into the NVB was considered as NVBI. SVI was evaluated as such with measurable infiltration of the SV (≥ 3 mm). SVC was present when the fat layer between the SV and the PCA lesion was no longer visible.

### Histopathologic reference standard

All patients underwent transrectal and hardware-assisted (DynaCAD, Philips, Invivo Corporation, USA) targeted MR/US fusion-guided biopsy and subsequent robotic-assisted radical prostatectomy (RPE). The intraoperatively obtained prostatic tissue and the tissue samples obtained by biopsy were examined according to the recommendations of the International Society of Urological Pathology (ISUP). Briefly, radical prostatectomy specimens were completely embedded after painting the surface using two colors to indicate the left and the right sides. The base and the apex were blocked separately. Whole-mount sections were not performed during the study period. ISUP Grade Groups from 1 to 5 (Gleason score 3 + 3 = 6, 3 + 4 = 7, 4 + 3 = 7, 4 + 4 = 8 or 4 + 5/5 + 4 = 9) were used for the final histopathological classification [21].

### Statistical analysis

Statistical analyses were conducted using IBM SPSS^®^ Statistics (Version 21, IBM Deutschland GmbH). Data are expressed as mean ± SD and median + IQR. Patient demographic data were reported using descriptive statistics. Performance of mpMRI was assessed by determining PPV, NPV, sensitivity, and specificity. Nonparametric Mann Whitney *U* test was used to compare two independent groups. Receiver operating characteristic (ROC) analyses were used for quantifying the impact of different predictors and Youden index (*J* = sensitivity + specificity − 1) to measure the clinical diagnostic ability. The maximum J was abbreviated as *J*_max_. Multivariate regression analysis was done to evaluate the relationship between T stage and predictive parameters. Statistical significance was defined as a *p* value < 0.05.

## Results

### Patient population

**Fig. 1:**
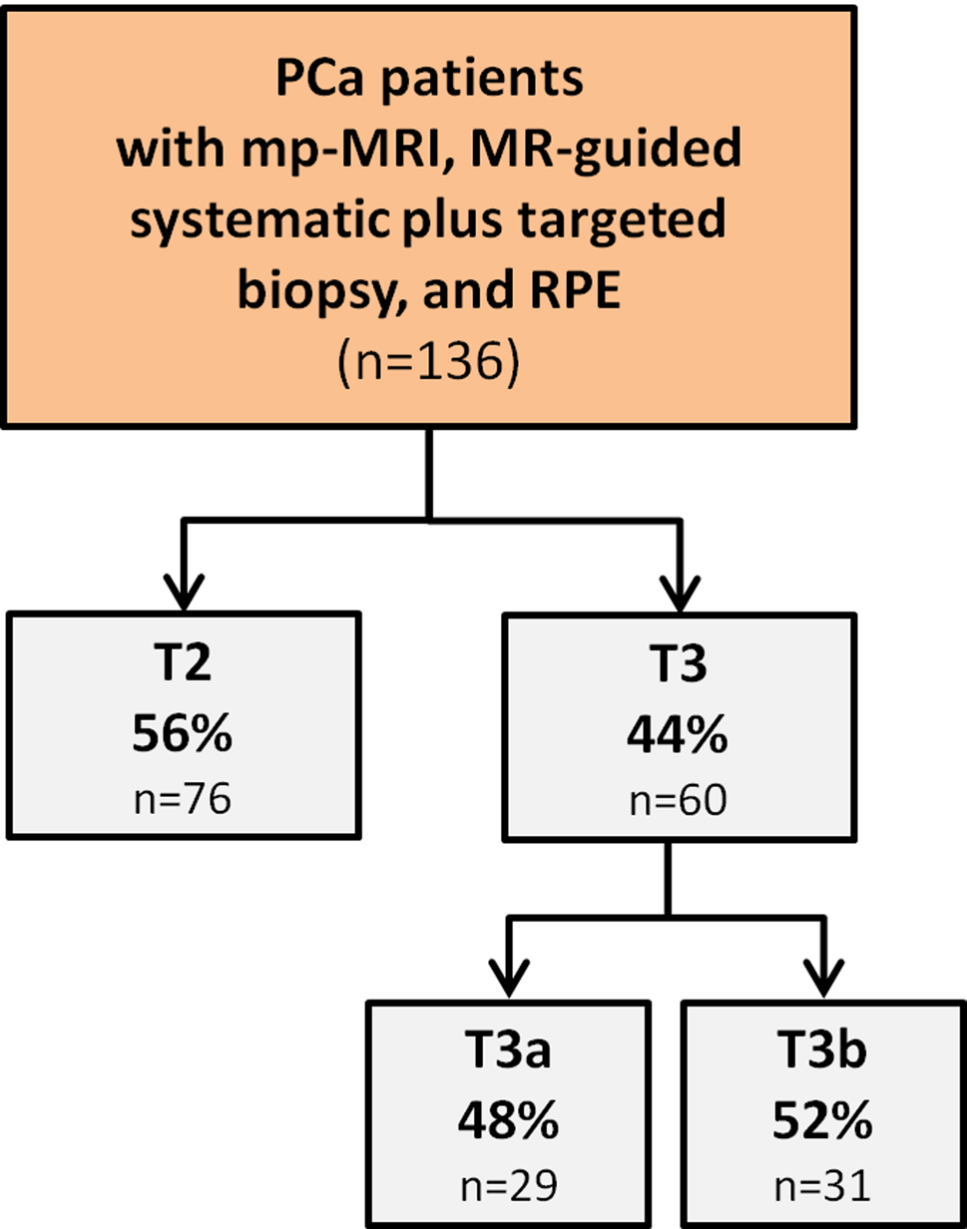
Flow chart of the patient collective

One hundred thirty six patients met the inclusion criteria. The mean age was 67 ± 5 years, the median PSA value was 9.3 (IQR 7.0–14) ng/ml, and the median PSAD was 0.25 ng/ml/ml. Patients received RPE 10 weeks after their MRI examination on average. T stage distribution is shown in Fig. 1, and baseline characteristics are illustrated in Table 1.

**Table 1:** Baseline characteristics

Patients number	136
Age years, median (IQR)	67 (62–72)
PSA ng/ml, median (IQR)	9.3 (7.0–14)
Prostate volume ml, median (IQR)	37 (30–50)
PSAD ng/ml/cm^3^, median (IQR)	0.25 (0.17–0.39)
ISUP grade group median (IQR)	
Post-biopsy	3 (2–4)
Post-surgery	3 (2–4)

*PSA* prostate-specific antigen, *PSAD* prostate-specific antigen density, *ISUP* International Society of Urological Pathology Grade Group

### T3 stage prediction

**Fig. 2:**
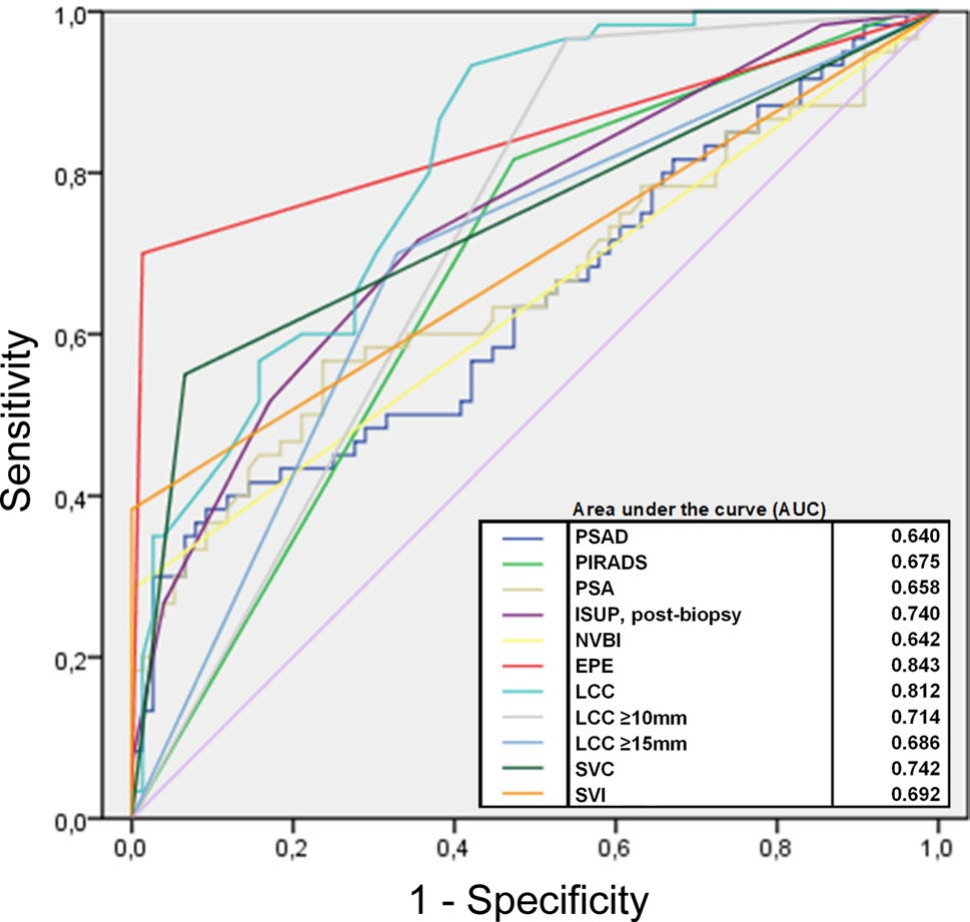
ROC analysis for T3 stage prediction

**Fig. 3:**
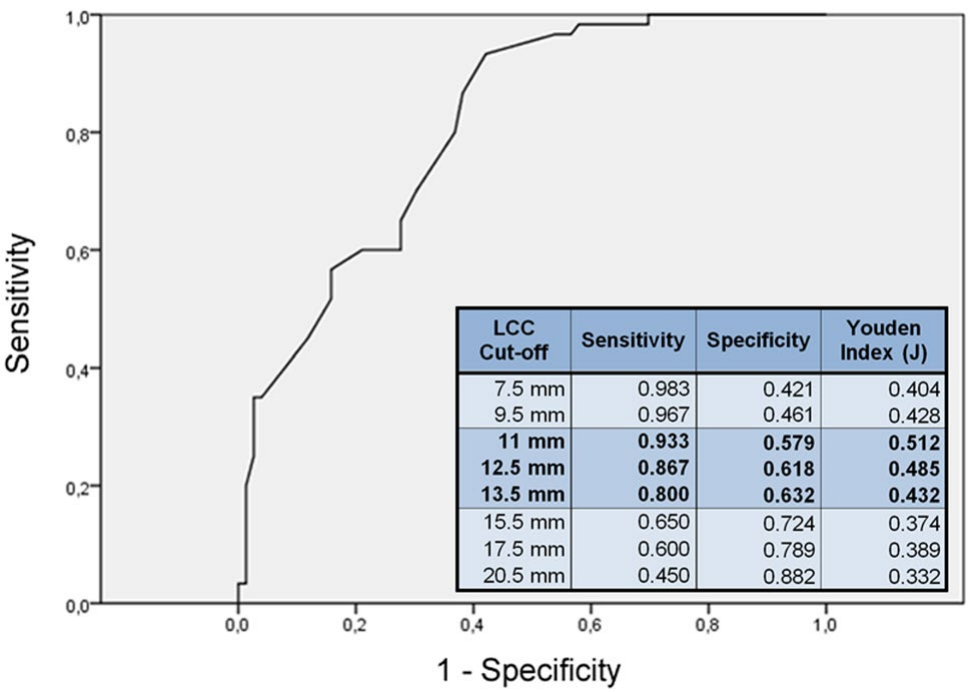
ROC analysis of the length of (pseudo)capsular contact (LCC) for T3 stage evaluation. Relevant LCC cutoff values are shown with corresponding sensitivity, specificity, and Youden index (J)

All 76 patients with a pT2 stage had significantly lower PSA, PSAD, lower PI-RADS scores, and post-biopsy ISUP Grade Group compared to those 60 patients with a pT3 stage. All predictors, including EPE, NVBI, SVC, SVI, and LCC, observed on MRI, were also significantly higher or more advanced in individuals with a pT3 stage. In one case, mpMRI misclassified a non-existing EPE. No visible or measurable NVBI or SVI was observed in patients with pT2 stage. Contact to the SV as well as the total LCC was significantly lower in pT2 compared to pT3 stages. All of the patients with histopathological-confirmed T3 stage were classified as PI-RADS 4 or 5. Mean lesion diameter for PI-RADS 5 lesions was 1.8 ± 4.5 cm and for PI-RADS 4 lesions 1.2 ± 2.4 cm. Detailed comparisons of patients with pT2 and pT3 stages are shown in Table 2. The ROC analysis showed that EPE (AUC 0.885) and LCC (AUC 0.812) were the best parameters to differentiate between pT2 and pT3 stages (Fig. 2, Supplementary Table 2). For T3 prediction a LCC threshold of 11 mm showed a sensitivity of 93% and specificity of 58% (*J* = 0.512), and a threshold of 12.5 mm showed 86% sensitivity and a specificity of 62% (*J* = 0.485). A threshold of 13.5 mm showed a sensitivity of 80% and a specificity of 63% (*J* = 0.432) (Fig. 3). A specificity of 88% and higher was achieved with LCC of ≥ 20.5 mm (*J* = 0.332). None of the pT3a stage cancer patients had a LCC of ≤ 5 mm. Multivariate analysis showed that the MRI parameters EPE and LCC were independent predictors in addition to the clinical parameters for the post-biopsy ISUP Grade Group (Table 3).

**Table 2:** Comparison of clinical and MRI parameter of patients with pT2 versus pT3 stage

	pT2	pT3	*p* value
Clinical			
Patients	76	60	
PSA ng/ml median (IQR)	8.8 (6.7–11)	12 (7.3–18)	< 0.01
PSAD ng/ml)/ml median (IQR)	0.23 (0.15–0.33)	0.28 (0.18–0.49)	< 0.01
ISUP, post-biopsy median (IQR)	2 (2–3)	4 (2–5)	< 0.001
MRI			
PI-RADS % (*n*)			
3	4 (3)	0	< 0.001
4	49 (37)	18 (11)	
5	47 (36)	82 (49)	
EPE % (*n*)	1.3 (1)	70 (42)	< 0.001
NVBI % (*n*)	0	28 (17)	< 0.001
SVC % (*n*)	7 (5)	55 (33)	< 0.001
SVI % (*n*)	0	38 (23)	< 0.001
LCC mm median (IQR)	10 (4–17)	20 (14–27)	< 0.001
LCC ≥ 10 mm % (*n*)	54 (41)	96 (58)	< 0.001
LCC ≥ 15 mm % (*n*)	33 (25)	70 (42)	< 0.001

*PSA* prostate-specific antigen, *PSAD* prostate-specific antigen density, *ISUP* International Society of Urological Pathology Grade Group, *EPE* extraprostatic extension, *NVBI* neurovascular bundle invasion, *SVC* seminal vesicle contact, *SVI* seminal vesicle infiltration, *LCC* length of pseudocapsular contact of tumor

**Table 3:** Multivariate regression analysis of T3 stage predictors

	*β*	*S*	OR	*p*	95% CI	
Clinical						
Age	− 0.07	0.05	0.94	0.17	0.85	10
PSAD	− 50	21	0.01	**0.04**	< 0.01	0.78
ISUP, post-biopsy	− 10	0.41	0.27	**< 0.01**	0.12	0.60
MRI						
PI-RADS	0.77	0.75	20	0.30	0.50	90
LCC	− 0.11	0.06	0.89	**0.05**	0.80	10
SVC	− 20	0.87	0.09	**0.01**	0.02	0.50
EPE	− 40	10	0.02	**< 0.01**	< 0.01	0.16

Bold values indicate statistically significant

*PSAD* prostate-specific antigen density, *ISUP* International Society of Urological Pathology Grade Group, *EPE* extraprostatic extension, *SVC* seminal vesicle contact, *LCC* length of pseudocapsular contact of tumor, *β* regressions coefficient, *S* standard error, *OR* odds ratio, *P p* value, *CI* confidence interval

### T3a and T3b discrimination

**Fig. 4:**
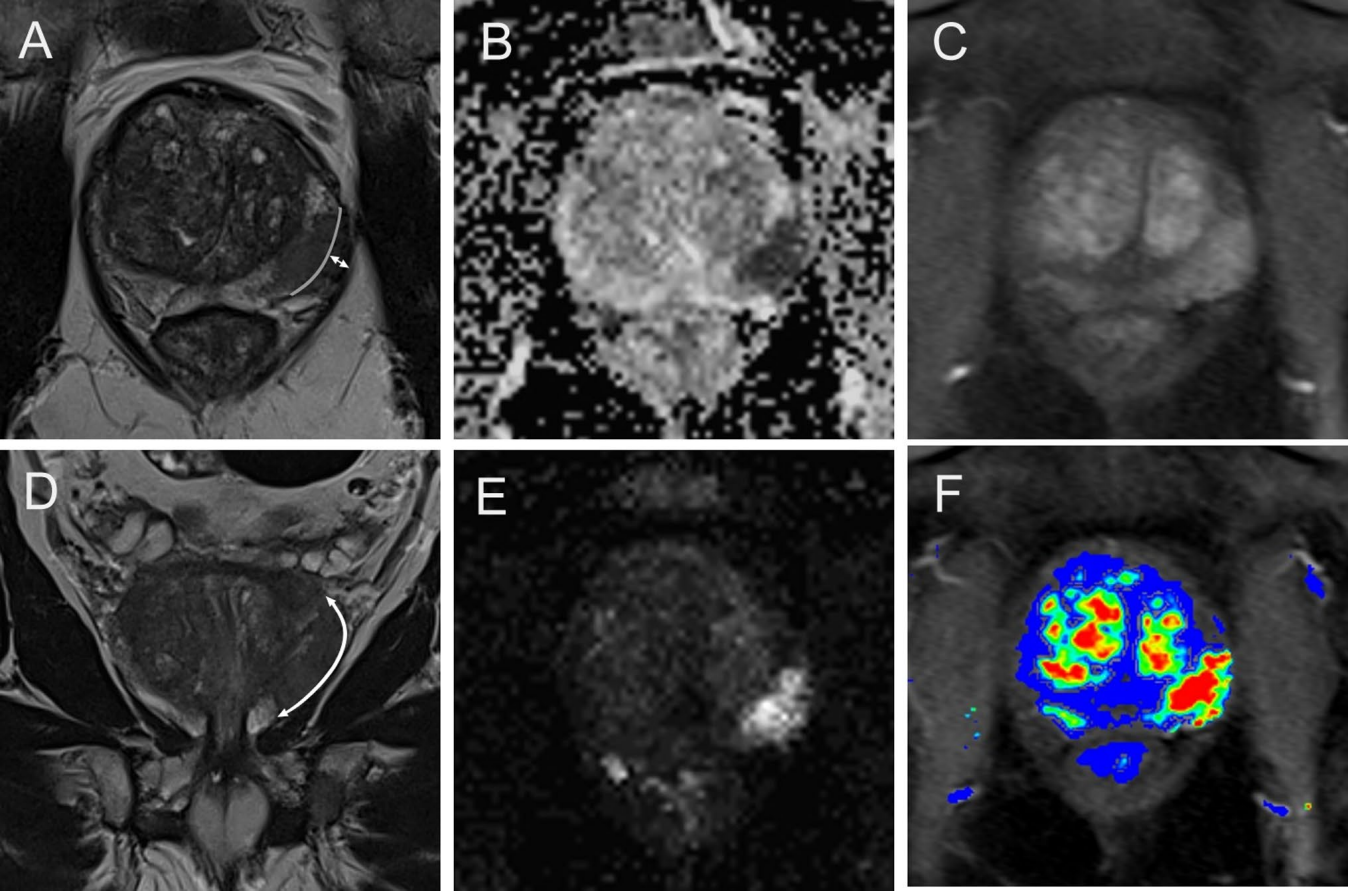
Example of T3a: **a** 72-year-old man with a PSAD of 0.24 ng/ml/ml. Axial (**a**) and coronal (**d**) T2W, readout-segmented, multishot EPI ADC (**b**), and high *b* value 1800 s/mm^2^ (**e**), DCE (**c**) and perfusion map (**f**) demonstrate a PCa suspicious lesion in the left peripheral zone. The lesion shows LCC of 21 mm (**b**, double-headed arrow) and measurable EPE of 4 mm (**a**, double-headed arrow), histopathologically confirmed as T3a stage, ISUP Grade Group 4

**Fig. 5:**
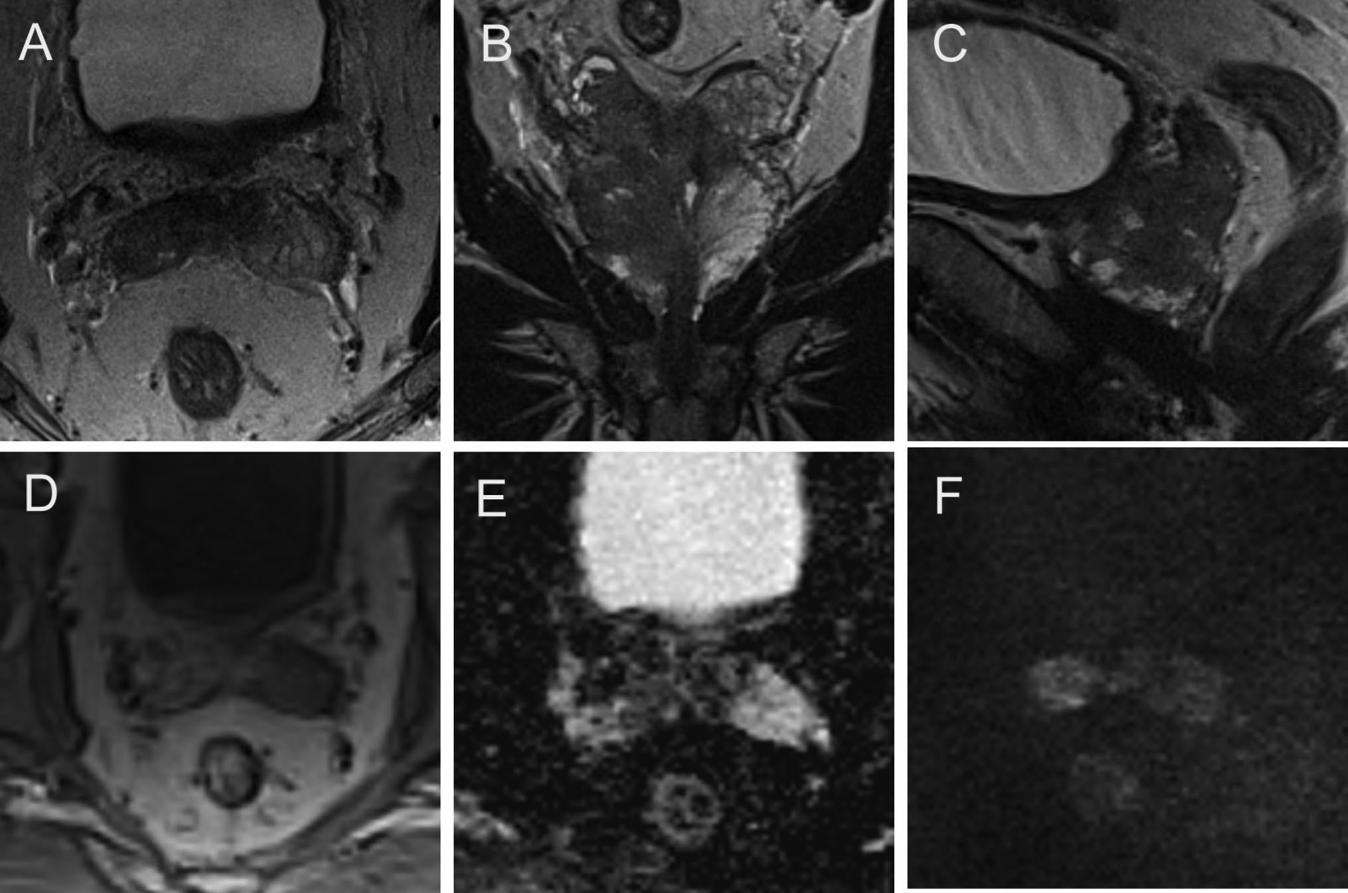
Example of T3b: **a** 73-year-old man with a PSAD of 0.23 ng/ml/ml. Axial (**a**), coronal (**b**), and sagittal (**c**) T2W, DCE (**d**), ADC (**e**), and DWI (**f**) demonstrate a lesion in the right peripheral and central gland SVI on both sides (right > left); histopathologically confirmed as T3b stage, ISUP Grade Group 3

The post-biopsy ISUP Grade Group, MRI-measurable EPE, SVC, or SVI was significantly different between pT3a and pT3b stage patients (Table 4). Forty-nine patients with a pT3a or pT3b stage (82%) were classified as PI-RADS 5. In 42 of the 60 patients with pT3 EPE (≥ 3 mm) was detected on MRI which derived a PPV of 98% (CI 0.88–1) and specificity of 99% (CI 0.93–1) for this descriptor. In 29 patients with pT3b a continuous contact of the PCA lesion to the SV was determined on MRI resulting in a sensitivity of 94% (CI 0.79–0.98) and a specificity of 91% (CI 0.85–0.95). Twenty-three patients demonstrated measurable SVI on MRI, and none of these patients had a T2 stage (PPV 100%, CI 0.86–1; specificity 100%, CI 0.97–1) (Table 5). A literature review regarding staging accuracy is shown in Supplementary Table 3 (Figs. 4, 5).

**Table 4:** Comparison of clinical and MRI parameter of patients with T3a versus T3b stage

	T3a	T3b	*p* value
Clinical			
Patients	29	31	
PSA ng/ml median (IQR)	13 (7.7–17)	11 (6.9–25)	0.95
PSAD ng/ml/ml median (IQR)	0.37 (0.18–0.49)	0.26 (0.2–0.49)	0.91
ISUP, post-biopsy median (IQR)	3 (2–4)	4 (3–5)	**0.01**
MRI			
PI-RADS % (*n*)			
4	21 (6)	16 (5)	0.65
5	79 (23)	84 (26)	
EPE % (*n*)	58 (17)	80 (25)	**0.02**
NVBI % (*n*)	20 (6)	35 (11)	0.21
SVC % (*n*)	14 (4)	94 (29)	**< 0.001**
SVI % (*n*)	0	74 (23)	**< 0.001**
LCC mm median (IQR)	18 (14–23)	21 (15–28)	0.09
LCC ≥15 mm % (*n*)	62 (18)	77 (24)	0.20

Bold values indicate statistically significant

*PSA* prostate-specific antigen, *PSAD* prostate-specific antigen density, *ISUP* International Society of Urological Pathology Grade Group, *EPE* extraprostatic extension, *NVBI* neurovascular bundle invasion, *SVC* seminal vesicle contact, *SVI* seminal vesicle infiltration, *LCC* length of capsular contact of tumor

**Table 5:** Accuracy of MRI for T3a or T3b stage prediction

Visibility on MRI		Sensitivity (CI 95%)	Specificity (CI 95%)	PPV (CI 95%)	NPV (CI 95%)
T3a	EPE	0.70 *(0.58*–*0.80)*	0.99 *(0.93*–*1.00)*	0.98 *(0.88*–*1.00)*	0.80 *(0.70*–*0.87)*
	NVBI	1.00 *(0.81*–*1.00)*	0.64 *(0.55*–*0.72)*	0.28 *(0.18*–*0.41)*	1.00 *(0.95*–*1.00)*
T3b	SVI	0.74 *(0.57*–*0.86)*	1.00 *(0.97*–*1.00)*	1.00 *(0.86*–*1.00*	0.93 *(0.87*–*0.96)*
	SVC	0.94 *(0.79*–*0.98)*	0.91 *(0.85*–*0.95)*	0.76 *(0.61*–*0.87)*	0.98 *(0.93*–*0.99)*

Italic values indicate CI 95%

*EPE* extraprostatic extension, *SVI* seminal vesicle infiltration, *NVBI* neurovascular bundle infiltration

## Discussion

According to the ESUR, mpMRI is currently the most useful method for local staging of PCA [22]. In this study, we reveal reliable predictors for the differentiation between a pT2 stage and a pT3 stage using 3 Tesla mpMRI. Our data show that measurable EPE > 3 mm and a LCC ≥ 11 mm were the best and independent predictors of a pT3 stage and measurable SVI can already confirm a pT3b stage.

We observed a high sensitivity of 93% for a histopathological T3 stage prediction in cases with a LCC of 11 mm and a sensitivity of 87% with a LCC of 12.5 mm. Results obtained by Dominguez et al. resulted with a LCC of 12 mm in a lower sensitivity of 69% for a potential invasion of the periprostatic tissue by using 1.5 Tesla mpMRI [10]. A LCC ≥ 20 mm was highly specific for the presence of a pT3 stage and none of the pT3 stage cases showed a LCC of ≤ 5 mm, i.e., in case of no or low LCC on MRI EPE is highly unlikely.

The medium sensitivity of 70% for detection of EPE using 3 Tesla mpMRI in our cohort is comparable to results by Feng et al. but higher than the reported 55% by Dominguez et al. or the 58% by Gaunay et al. [10, 23, 24]. However, Feng et al. showed a lower accuracy of mpMRI to predict the presence of EPE in the prostatic apex. Baco et al. were able to improve the specificity for detection of EPE by adding indirect signs like capsule protrusion and LCC higher than 20 mm. This increased specificity from 57% to 85% [25]. However, sensitivity would be decreased by a higher cutoff and pT3 tumors with less than 20 mm LCC may be misclassified.

Regarding the prediction of NVBI, we found similar results to studies by other groups [26]. However, a stage T3a does not entirely preclude nerve-sparing surgery. Often, contralateral nerves can be spared, and an ipsilateral partial nerve-sparing surgery can be undertaken depending on the degree of extraprostatic extension. In terms of sensitivity for measurable SVI, we achieved better results than Roethke et al. (48%), who used a 1.5 Tesla MRI with an endorectal coil [19]. This may be explained by the higher field strength of 3 Tesla, better image quality, and increased performance of DWI [27]. Correspondingly, compared to groups that also used a 3 Tesla MRI, we were able to achieve similar results for the prediction of SVI [18]. In addition to the lower field strength, post-inflammatory changes in the SV, such as wall thickening or collapsed SV can also result in lower sensitivity.

Currently, different predictive models such as CAPRA score, D’Amico risk groups, Partin staging tables, Kattan nomograms, or Classification and Regression Tree (CART) analysis are used in clinical routine to assess the individual risk of patients with newly diagnosed PCA. Results of these predictive models are essential for the treatment decision-making process [8, 28–31]. Additional predictors for a pT3 stage such as imaging modalities offer the possibility of a more precise, accurate, and non-invasive preoperative T assessment, which provides the surgeon with detailed information on hand for appropriate therapy. Besides LCC, 3 Tesla mpMRI offers information about the tumor morphology and localization [32]. Thus, preoperative boundaries for frozen section preparations could be defined, especially in areas where the tumor approaches the capsule or the NVB. Since 3 Tesla mpMRI shows higher sensitivity, for example, for LCC compared to 1.5 Tesla mpMRI, it must be stated that only 3 Tesla mpMRI guarantees an accurate, predictive method for preoperative stage assessment [33]. Also, 3 Tesla mpMRI provides a significant improvement compared to 1.5 Tesla mpMRI even without the use of an endorectal coil [35]. In addition, the ability to predict the preoperative tumor stage can be used to decide if adjuvant radiation is needed or can influence the surgical strategy like side-specific nerve-sparing resection, bladder neck dissection or extension of lymphadenectomy [33–35]. For example, the preservation of the NVB allows better functional outcomes with respect to erectile function, which has a high impact on the quality of life. In addition to that, the oncological outcomes such as remaining tumor residuals can be improved with higher recurrence free rates as well as higher cancer-specific survival. Thus, prediction of the T3 stage using 3 Tesla mpMRI could complement and improve the preoperative assessment.

This study has limitations. Next to the retrospective design and the single-center evaluation, the relatively small number of patients at risk, and the time interval between MRI and RPE may have influenced the results. However, the average time interval between mpMRI and surgery was only 11 weeks, and prostate cancers usually grow slowly compared to other tumor entities.

In conclusion, 3 Tesla mpMRI with a phased-array surface coil allows accurate PCA tumor stage assessment. MRI measurable EPE, LCC, and SVC of the PCA lesion were reliable, independent predictors of a pT3 stage next to visible invasions of NVB. SVC furthermore strongly correlates with a pT3b stage and measurable infiltration definitely confirms this T status. Therefore, MRI enables accurate, individual therapy planning (nerve-sparing, extended surgery, radiation therapy, etc.), and pre-biopsy MRI can improve functional and oncologic patient outcomes.

## Supplementary information

Below is the link to the electronic supplementary material.

Supplementary material 1 (DOCX 15 kb)

Supplementary material 3 (DOCX 13 kb)

Supplementary material 2 (DOCX 18 kb)

## Abbreviations

CAPRA: Cancer of the prostate risk assessment
LCC: Length of (pseudo)capsular contact
DCE: Dynamic contrast-enhanced imaging
DWI: Diffusion-weighted imaging
EPE: Extraprostatic extension
FEPE: Focal/microscopic extraprostatic extension
ISUP: International Society of Urological Pathology
mpMRI: Multiparametric magnetic resonance imaging
NPV: Negative predictive value
NVBI: Invasion of neurovascular bundle
PCA: Prostate cancer
PI-RADS: Prostate imaging reporting and data system
PPV: Positive predictive value
PSA: Prostate-specific antigen
PSAD: PSA density
RPE: Radical prostatectomy
SVC: Seminal vesicles lesion contact
SVI: Seminal vesicles infiltration
T2WI: T2-weighted imaging
*J*_max_: Maximum Youden index (*J*)
