# Supplementary material for: Magnetic resonance imaging improves the prediction of tumor staging in localized prostate cancer
Source: Abdom Radiol (NY). 2021 Jan 16;46(6):2751–9. doi: 10.1007/s00261-020-02913-9 (PMC8205913; doi:10.1007/s00261-020-02913-9)
Supplement: Supplementary file 1 — Supplementary material 1 (DOCX 15 kb) [file 261_2020_2913_MOESM1_ESM.docx]

**Supplemental Table 1:** MRI protocols.

| **3T Magnetom Skyra®** (Siemens Healthcare GmbH) | | | | | | | |
| --- | --- | --- | --- | --- | --- | --- | --- |
| **Sequence** | T2 TSE | | | rs-EPI  DWI | ss-EPI DWI | T1 TSE | T1 Vibe DCE |
| **Orientation** | Sagittal | Coronal | Axial | Axial | Axial | Axial | Axial |
| **TR (ms)** | 7870 | 7430 | 8770 | 6200 | 7800 | 712 | 3.90 |
| **TE (ms)** | 98 | 98 | 79 | 56/84 | 88 | 13 | 1.39 |
| **Matrix size** | 320 | 320 | 256 | 112 | 136 | 576 | 256 |
| **Thickness(mm)** | 3 mm | 3 mm | 3 mm | 3 mm | 3 mm | 5 mm | 3 mm |
| **Voxel size (mm)** | 0.5x0.5x3 | 0.5x0.5x3 | 0.5x0.5x3 | 1.8x1.8x3 | 1.5x1.5x3 | 0.6x0.6x5 | 0.8x0.8x3 |
| **FOV** | 170 | 170 | 130 | 200 | 200 | 350 | 200 |
| **b-values (sec/mm2)** | - | - | - | 0, 1000  + 1600 (calculated) | 0, 500, 1000  + 1500 (calculated) | - | - |
| **Contrast agent** | - | - | - | - | - | - | 0.1 mmol/Kg gadolinium |
| **Temporal resolution** | - | - | - | - | - | - | 7 sec. |

| **3T Magnetom Trio®** (Siemens Healthcare GmbH) | | | | | | |
| --- | --- | --- | --- | --- | --- | --- |
| **Sequence** | T2 TSE | | | ss-EPI DWI | T1 TSE | T1 Vibe DCE |
| **Orientation** | Sagittal | Coronal | Axial | Axial | Axial | Axial |
| **TR (ms)** | 11330 | 11330 | 10630 | 4700 | 650 | 3.62 |
| **TE (ms)** | 103 | 103 | 117 | 90 | 13 | 1.27 |
| **Matrix size** | 256 | 256 | 256 | 136 | 320 | 128 |
| **Thickness(mm)** | 3 mm | 3 mm | 3 mm | 3 mm | 5 mm | 3 mm |
| **Voxel size (mm)** | 0.7x0.7x3 | 0.7x0.7x3 | 0.5x0.5x3 | 1.5x1.5x3 | 1.3x0.9x5 | 1.5x1.5x3.3 |
| **FOV** | 170 | 170 | 128 | 200 | 300 | 192 |
| **b-values (sec/mm2)** | - | - | - | 0, 500,  1000  + 1400 (acquired) | - | - |
| **Contrast agent** | - | - | - | - |  | 0.1 mmol/Kg gadolinium |
| **Temporal resolution** | - | - | - | - | - | 9 sec. |
