# Supplementary material for: Magnetic resonance imaging improves the prediction of tumor staging in localized prostate cancer
Source: Abdom Radiol (NY). 2021 Jan 16;46(6):2751–9. doi: 10.1007/s00261-020-02913-9 (PMC8205913; doi:10.1007/s00261-020-02913-9)
Supplement: Supplementary file 2 — Supplementary material 3 (DOCX 13 kb) [file 261_2020_2913_MOESM2_ESM.docx]

**Supplemental Table 2:** Literature review of MRI prostate staging accuracy.

| **Author** | **Journal, year** | **Titel** | **Study design** | **Findings** |
| --- | --- | --- | --- | --- |
| Roethke et al. | Urol Int, 2014 | Seminal vesicle invasion: accuracy and analysis of infiltration patterns with high-spatial resolution T2-weighted sequences on endorectal magnetic resonance imaging. | n=376  retrospective design  1.5 Tesla MRI scanner | SVI (sensitivity): 48% |
| Feng et al. | Urol Oncol, 2015 | Multiparametric magnetic resonance imaging localizes established extracapsular extension of prostate cancer. | n=112  retrospective design  3 Tesla MRI scanner | EPE (sensitivity): 70% |
| Baco et al. | J Urol, 2015 | Predictive value of magnetic resonance imaging determined tumor contact length for extracapsular extension of prostate cancer. | n=111  retrospective design | Cut of LCC = 20 mm  EPE (sensitivity): 82% |
| de Rooij et al. | Eur Urol, 2016 | Accuracy of Magnetic Resonance Imaging for Local Staging of Prostate Cancer: A Diagnostic Meta-analysis. | n= 9796  metaanalysis  1.0 to 3.0 Tesla MRI scanner | EPE (sensitivity): 57%  SVI (sensitivity): 58%  T3 disease (sensitivity): 61% |
| Gaunay et al. | Asian J Urol, 2017 | Multi-parametric MRI of the prostate: Factors predicting extracapsular extension at the time of radical prostatectomy | n=1722  prospective design  3 Tesla MRI scanner | ECE (sensitivity): 58% |
| Dominguez et al. | Int Braz J Urol, 2018 | Diagnostic accuracy of multiparametric magnetic resonance imaging in detecting extracapsular extension in intermediate and high - risk prostate cancer | n=79  retrospective  1.5 Tesla MRI scanner | EPE (sensitivity): 54%  SVI (sensitivity): 19% |
| Grivas et al. | Eur J Radiol, 2018 | Seminal vesicle invasion on multi-parametric magnetic resonance imaging: Correlation with histopathology. | n=527  retrospective design  3 Tesla MRI scanner | SVI (sensitivity): 75% |

*n= number of patients; EPE: extraprostatic extension; SVI: seminal vesicle infiltration; LCC: length capsular contact of the tumor*
