# Supplementary material for: Magnetic resonance imaging improves the prediction of tumor staging in localized prostate cancer
Source: Abdom Radiol (NY). 2021 Jan 16;46(6):2751–9. doi: 10.1007/s00261-020-02913-9 (PMC8205913; doi:10.1007/s00261-020-02913-9)
Supplement: Supplementary file 3 — Supplementary material 2 (DOCX 18 kb) [file 261_2020_2913_MOESM3_ESM.docx]

**Supplemental Table 3:** ROC analysis of T3 stage predictors.

|  | | **AUC** | **S** | **95% CI** | | **P** |
| --- | --- | --- | --- | --- | --- | --- |
|  | **PSA** (ng/ml) | 0.658 | 0.049 | 0.562 | 0.753 | **0.002** |
|  | **PSAD** (ng/ml)/ml) | 0.640 | 0.049 | 0.545 | 0.736 | **0.005** |
|  | **ISUP, post-biopsy** | 0.740 | 0.043 | 0.657 | 0.824 | **0.000** |
| **MRI** | **PI-RADS** | 0.675 | 0.046 | 0.585 | 0.765 | **0.000** |
|  | **EPE** | 0.885 | 0.029 | 0.828 | 0.942 | **0.000** |
|  | **FEPE** | 0.843 | 0.038 | 0.769 | 0.918 | **0.000** |
|  | **SVC** | 0.755 | 0.044 | 0.668 | 0.842 | **0.000** |
|  | **SVI** | 0.692 | 0.048 | 0.598 | 0.785 | **0.000** |
|  | **LCC** (mm) | 0.812 | 0.036 | 0.742 | 0.882 | **0.000** |
|  | **LCC ≥10 mm** | 0.714 | 0.044 | 0.628 | 0.799 | **0.000** |
|  | **LCC ≥15 mm** | 0.686 | 0.046 | 0.595 | 0.776 | **0.000** |

*PSA = prostate specific antigen; PSAD = prostate specific antigen density; EPE = extraprostatic extension; FEPE = focal/microscopic extraprostatic extension; NVBI = neurovascular bundle invasion; SVC = seminal vesicle contact; SVI = seminal vesicle infiltration; LCC = length of pseudocapsular contact of tumor; ISUP = International Society of Urological Pathology Grade Group; AUC = Areal under the curve; S = standard error; P = p value; CI = confidence interval*
